# Novel Biomaterials for Tissue Engineering

**DOI:** 10.3390/jfb17050231

**Published:** 2026-05-07

**Authors:** Mina Aleemardani, Farnaz Ghorbani

**Affiliations:** 1 Department of Translational Health Science, Bristol Medical School, University of Bristol, Bristol BS1 3NY, UK; 2 Institute of Orthopaedic & Musculoskeletal Science, University College London, Royal National Orthopaedic Hospital, Stanmore HA7 4LP, UK

## 1. Introduction

Recent years have seen significant progress in functional biomaterials for biomedical applications, driven by advances in materials engineering, nanotechnology, and biological characterization techniques. These developments have improved the design materials with enhanced biological performance and therapeutic potential, opening new opportunities in regenerative medicine. However, a key translational bottleneck remains, as results from simplified in vitro systems often fail to reliably predict in vivo behavior, limiting clinical translation. Challenges persist in achieving precise control over biomaterial–biological interactions and in bridging the gap between laboratory research and clinical application [1,2,3].

This Special Issue, **Novel Biomaterials for Tissue Engineering**, brings together six contributions focused on biomaterials that not only support tissue repair but also actively interact with and regulate biological responses. Rather than simply presenting different material systems, the issue highlights a shift toward bioinstructive materials that better engage with the native cellular microenvironment to improve regenerative outcomes. Accordingly, tissue engineering continues to evolve as an interdisciplinary field at the interface of materials science, biology, and engineering, where recent advances in scaffold design increasingly enable closer mimicry of the extracellular matrix and improved control over key cellular functions such as attachment, proliferation, and differentiation.

The articles included in this Special Issue highlight the diversity of biomaterials being explored for tissue engineering applications, including natural polymers, marine-derived materials, nanostructured systems, and clinically relevant biomaterial platforms. Collectively, they emphasize that biomaterial performance should be evaluated within a biological context, and that careful biological assessment is essential to ensure the safety, functionality, and translational potential of emerging biomaterials for biomedical applications.

## 2. Special Issue Highlights

The earliest contribution in this Special Issue by Berry-Kilgour et al. investigates the potential of marine macroalgae as a sustainable source of cellulose scaffolds for tissue engineering applications. Using a chemical decellularization process, the authors produced cellulose matrices from several macroalgae species while preserving their natural structural architecture. Their results showed that scaffolds derived from Ecklonia radiata supported fibroblast attachment and maturation, highlighting their potential for skin tissue engineering. This work reflects the growing interest in marine-derived biomaterials as environmentally sustainable alternatives for biomedical scaffold fabrication [4].

In another study, Mahmoud et al. explore the biological effects of bovine serum albumin (BSA)-coated gold nanorods on human dermal fibroblasts. The authors assessed cell viability, migration, and the production of inflammatory mediators following nanoparticle exposure. Although the nanorods exhibited relatively low cytotoxicity across a range of concentrations, the study revealed notable effects on cellular behavior, including delayed wound closure in migration assays. These findings highlight the importance of evaluating functional cellular responses, in addition to cytotoxicity, when developing nanoparticle-based biomaterials for regenerative applications [5].

A comprehensive review by Premjit et al. provides an overview of biomimetic three-dimensional scaffolds derived from sustainable natural biomaterials for bone tissue engineering. The authors summarize recent advances in scaffold fabrication using plant- and animal-derived polymers that mimic the structural and biochemical characteristics of the native extracellular matrix. The review also discusses emerging strategies involving nanotechnology, antimicrobial approaches, and advanced fabrication techniques aimed at enhancing scaffold functionality and promoting bone regeneration [6].

Another review article by Sierra-Sanchez et al. examines the use of blood plasma-, fibrinogen-, and fibrin-based biomaterials in skin tissue engineering and wound healing applications. Through a systematic analysis of preclinical and clinical studies, the authors evaluated the use of these materials as wound dressings and skin substitutes. Their findings indicate that plasma-derived biomaterials exhibit high biocompatibility and can effectively support wound healing processes. However, the authors also identify several limitations in the current literature, including the lack of standardized experimental models and limited long-term clinical evidence [7].

In a more recent experimental study, Kang et al. investigated an epigallocatechin gallate (EGCG)-loaded hyaluronic acid hydrogel in a rat model of collagenase-induced Achilles tendinopathy. Histological, immunofluorescence, and molecular analyses showed increased type I collagen expression with reduced apoptosis and inflammatory markers. These results suggest that antioxidant-loaded hydrogels may support tendon regeneration. However, further work is needed to address translational challenges, including long-term in vivo performance, scalable fabrication, and validation in clinically relevant models before clinical application [8].

The most recent contribution by Sun et al. presents an in vitro blood–brain barrier (BBB) micro-organoid model using a decellularized squid mantle scaffold film. The study addresses limitations of existing BBB models that poorly replicate native structural complexity and cellular interactions. The marine-derived scaffold created a biomimetic microenvironment supporting relevant cellular organization. The resulting model showed promising structural and functional features, suggesting potential applications of marine-derived biomaterials in advanced in vitro systems for neurological research, drug screening, and disease modeling [9].

## 3. Concluding Perspective

Taken together, the contributions included in this Special Issue highlight the breadth of current research in novel biomaterials for tissue engineering, spanning marine-derived scaffolds, nanoparticle-based systems, biomimetic matrices, and clinically oriented biomaterials. While this diversity reflects the vibrancy of the field, it also highlights underscores a key challenge: many promising materials remain at the proof-of-concept or early preclinical stage, with limited progress toward standardized evaluation and clinical translation.

Moving forward, greater emphasis is needed on translationally driven biomaterial design, where biological performance is assessed in models that better reflect human physiology. This includes wider use of advanced in vitro systems (such as organoids and organ-on-chip platforms), alongside robust in vivo validation and standardized benchmarking. Overall, progress toward clinical application will depend not only on interdisciplinary collaboration but also on improved scalability, reproducibility, and regulatory alignment in biomaterial development.

The Guest Editors would like to thank all authors for their valuable contributions and the reviewers for their thoughtful and constructive feedback. We also extend our appreciation to the Editorial Office for their support throughout the publication process. We hope that this Special Issue will inspire further research and foster continued collaboration in this rapidly evolving field.

## References

[1] Wang M., Hong Y., Fu X., Sun X. (2024). Advances and Applications of Biomimetic Biomaterials for Endogenous Skin Regeneration. Bioact. Mater..

[2] Holmes T.C. (2002). Novel Peptide-Based Biomaterial Scaffolds for Tissue Engineering. Trends Biotechnol..

[3] Khan H.M., Liao X., Sheikh B.A., Wang Y., Su Z., Guo C., Li Z., Zhou C., Cen Y., Kong Q. (2022). Smart Biomaterials and Their Potential Applications in Tissue Engineering. J. Mater. Chem. B.

[4] Berry-Kilgour C., Oey I., Cabral J., Dowd G., Wise L. (2024). Decellularized Green and Brown Macroalgae as Cellulose Matrices for Tissue Engineering. J. Funct. Biomater..

[5] Mahmoud N.N., Hammad A.S., Al Kaabi A.S., Alawi H.H., Khatoon S., Al-Asmakh M. (2024). Evaluating the Effects of BSA-Coated Gold Nanorods on Cell Migration Potential and Inflammatory Mediators in Human Dermal Fibroblasts. J. Funct. Biomater..

[6] Premjit Y., Lawrence M., Goyal A., Ferreira C., Jones E.A., Ganguly P. (2025). Biomimetic Three-Dimensional (3D) Scaffolds from Sustainable Biomaterials: Innovative Green Medicine Approach to Bone Regeneration. J. Funct. Biomater..

[7] Sierra-Sánchez Á., Sanabria-de la Torre R., Ubago-Rodríguez A., Quiñones-Vico M.I., Montero-Vílchez T., Sánchez-Díaz M., Arias-Santiago S. (2025). Blood Plasma, Fibrinogen or Fibrin Biomaterial for the Manufacturing of Skin Tissue-Engineered Products and Other Dermatological Treatments: A Systematic Review. J. Funct. Biomater..

[8] Kang H.J., Subramanian S.A., Song S.Y., Hwang J., Lee C., Kim S.J. (2025). Epigallocatechin-3-Gallate (EGCG)-Loaded Hyaluronic Acid Hydrogel Seems to Be Effective in a Rat Model of Collagenase-Induced Achilles Tendinopathy. J. Funct. Biomater..

[9] Sun H., Diao X., Feng J., Wang H., Elango J., Wu W. (2026). Construction of an In Vitro Blood–Brain Barrier Micro-Organoid Model Using Decellularized Squid Mantle Scaffold Film. J. Funct. Biomater..

